# How to involve the younger generation in the European Public Health Week? Monica Brînzac

**DOI:** 10.1093/eurpub/ckac129.287

**Published:** 2022-10-25

**Authors:** M Brînzac

**Affiliations:** EUPHAnxt

## Abstract

This presentation will tell you about the involvement of the next generation: young public health professionals in the European Public Health Week.

